# 576. A Peer-supported Guide for Firefighters Healing from Burn Injury: Bridging Survivor Experience and Clinical Expertise

**DOI:** 10.1093/jbcr/irag033.339

**Published:** 2026-04-06

**Authors:** Monica Hutson, Darren Jones, Michael Erickson

**Affiliations:** The University of Texas Medical Branch Blocker Burn Unit, Galveston, TX; Houston Fire Department, Houston, TX; Blocker Burn Unit, Kemah, TX

## Abstract

**Introduction:**

Firefighters who sustain burn injuries face unique physical, psychological, and social challenges during recovery. Despite the prevalence of line-of-duty burn injuries, few resources are tailored to help firefighters and their families through the continuum of care. This project aimed to bridge that gap by developing The Burn Recovery Red Book: A Peer-Supported Guide for Firefighters Healing from Injury, which combines lived experience, peer support, and clinical expertise into a structured recovery framework.

**Methods:**

A qualitative, collaborative process was employed. The lead author, a firefighter who experienced a burn injury, engaged in reflective journaling and narrative reconstruction of his recovery. Peer Support Team members from the Fire Department and clinicians from the burn center participated in semi-structured dialogues. These discussions examined all phases of care, from on-scene response and initial management to long-term reintegration, focusing on what facilitated recovery and what barriers existed. Insights were iteratively synthesized into patient- and peer-centered guidance.

**Results:**

The collaboration produced a comprehensive guide addressing immediate response, initial medical management, inpatient burn center care, preparing for home recovery, rehabilitation, and reintegration into duty. Key findings highlighted the critical role of peer validation and the importance of clear and accessible patient education. Peer Support Team members reported increased confidence in addressing burn recovery, while clinicians gained a deeper understanding of firefighter culture and recovery needs.

**Conclusions:**

This project demonstrated the value of integrating lived experience with clinical insight to create a recovery guide tailored to the unique needs of firefighters healing from burn injuries. The collaborative process revealed areas for improvement in burn recovery and highlighted the importance of peer-informed, culturally relevant education. The resulting guide offers a practical, empathetic framework that supports both survivors and those who care for them, reinforcing the role of peer support as a vital component of recovery.

**Applicability of Research to Practice:**

This collaborative framework highlights how peer-supported, experience-informed education can improve burn recovery in firefighters by enhancing outcomes, easing psychological strain, and supporting reintegration.

**Funding for the study:**

N/A.

